# Genetic Association of Human Leukocyte Antigens with Chronicity or Resolution of Hepatitis B Infection in Thai Population

**DOI:** 10.1371/journal.pone.0086007

**Published:** 2014-01-23

**Authors:** Nawarat Posuwan, Sunchai Payungporn, Pisit Tangkijvanich, Shintaro Ogawa, Shuko Murakami, Sayuki Iijima, Kentaro Matsuura, Noboru Shinkai, Tsunamasa Watanabe, Yong Poovorawan, Yasuhito Tanaka

**Affiliations:** 1 Department of Virology and Liver Unit, Nagoya City University Graduate School of Medical Sciences, Nagoya, Japan; 2 Research Unit of Hepatitis and Liver Cancer, Faculty of Medicine, Chulalongkorn University, Bangkok, Thailand; 3 Department of Biochemistry, Faculty of Medicine, Chulalongkorn University, Bangkok, Thailand; 4 Center of Excellence in Clinical Virology, Faculty of Medicine, Chulalongkorn University, Bangkok, Thailand; The University of Hong Kong, Hong Kong

## Abstract

**Background:**

Previous studies showed that single nucleotide polymorphisms (SNPs) in the *HLA-DP*, *TCF19* and *EHMT2* genes may affect the chronic hepatitis B (CHB). To predict the degree of risk for chronicity of HBV, this study determined associations with these SNPs.

**Methods:**

The participants for this study were defined into 4 groups; HCC (n = 230), CHB (n = 219), resolved HBV infection (n = 113) and HBV uninfected subjects (n = 123). The *HLA-DP* SNPs (rs3077, rs9277378 and rs3128917), *TCF19* SNP (rs1419881) and *EHMT2* SNP (rs652888) were genotyped.

**Results:**

Due to similar distribution of genotype frequencies in HCC and CHB, we combined these two groups (HBV carriers). The genotype distribution in HBV carriers relative to those who resolved HBV showed that rs3077 and rs9277378 were significantly associated with protective effects against CHB in minor dominant model (OR = 0.45, *p*<0.001 and OR = 0.47, *p*<0.001). The other SNPs rs3128917, rs1419881 and rs652888 were not associated with HBV carriers.

**Conclusions:**

Genetic variations of rs3077 and rs9277378, but not rs3128917, rs1419881 and rs652888, were significantly associated with HBV carriers relative to resolved HBV in Thai population.

## Introduction

The hepatitis B virus (HBV) is one of the most common causes of chronic hepatitis B (CHB), liver cirrhosis and hepatocellular carcinoma (HCC). Globally more than 2 billion people have been infected with HBV and 378 million are suffering from chronic hepatitis. Over 600,000 people die each year because of HBV infection. In high prevalence areas such as the central Asian republics, Southeast Asia, Sub-Saharan Africa and the Amazon basin over 8% of the population may be HBV carriers [1]. The main route of HBV infection is vertical transmission from mother to infant and horizontal transmission between children, whereby 90% will develop chronic hepatitis as infants or in early childhood and never clear the virus [1]–[3]. In contrast, 15% of HBV infections in adulthood develop into chronic hepatitis with viral persistence.

The frequency of HBV infection which develops into chronic hepatitis depends on the age at which the person is infected [1], [2]. However, the factors determining HBV persistence or clearance are not clearly understood [4]–[6]. Risk factors for viral persistence include the following: virological factors (viral load, genotype, viral gene mutations and co-infection with another virus), host factors (age at infection, gender, immune status and genetic variability) and extrinsic factors (e.g. alcohol consumption and chemotherapy) [7]. Whether viral infection results in acute or chronic infection also depends on cellular immune responses influenced by human leukocyte antigen (*HLA*) class I and II molecules which must present the viral antigens to CD8+ T cells and CD4+ T cells, respectively [8]. The genes encoding *HLA* are the most polymorphic in the human genome, presumably in order to be able to respond to all potential foreign antigens [9].

Recently, many genome-wide association studies (GWAS) have been performed to seek associations between human genetic variation and the outcome of HBV infection [10]–[15]. Studies in the Japanese population showed that 11 single nucleotide polymorphisms (SNPs) located within or around the *HLA-DPA1* and *HLA-DPB1* loci are significantly associated with the occurrence of CHB. Of these 11 SNPs, the most strongly associated with the outcome of HBV infection were rs9277535 and rs3128917 in *HLA-DPB1* and rs3077 in *HLA-DPA1* [10].

Thereafter, GWAS studies in the Korean population confirmed the presence of these host factors related to HBV outcome and reported two new SNPs significantly associated with CHB within the *HLA* region, namely rs1419881 and rs652888 in transcription factor 19 (*TCF19*) and euchromatic histone-lysine methyltransferase 2 (*EHMT2*), respectively [16]. *TCF19* (or transcription factor SC1) is a *trans*-activating factor that mainly influences the transcription of genes required for late growth regulation at the G1-S checkpoint and during S phase [17]. *EHMT2* is a histone methyltransferase responsible for mono- and di-methylation of H3K9 (lysine at 9^th^ residue of histone subunit 3) in euchromatin [18], which modifies the conformation of chromatin from its tightly packed form, heterochromatin, and thus influences gene repression or transcriptional silencing [19].

In the present study, we determined associations between the SNPs of *HLA-DPA1* (rs3077), *HLA-DPB1* (rs9277378 and rs3128917), *TCF19* (rs1419881) and *EHMT2* (rs652888) in HBV infected patients compared to those with resolved infections and those who had never been infected.

## Materials and Methods

### Ethics Statement

This study was approved by the Institutional Review Board of the Faculty of Medicine, University (Bangkok, Thailand) code IRB.455/54. Written informed consent was obtained from each patient and all samples were anonymized.

### Sample Collection

All blood samples were negative for hepatitis C virus and human immunodeficiency virus. Subjects were defined into 4 groups: 230 hepatitis B surface antigen (HBsAg)-positive HCC, and 219 CHB who had been HBsAg-positive for at least 6 months were recruited at the King Chulalongkorn Memorial Hospital, whereas patients with resolved HBV and uninfected subjects were from the Thai Red Cross Society and from the north-eastern part of Thailand (age>40 years) which had been screened by Immunoassay (Architect i2000SR, Abbott, USA.) for HBsAg, antibody to hepatitis B surface antigen (anti-HBs) and antibody to hepatitis B core protein (anti-HBc). Of these subjects, 113 were negative for HBsAg but positive for anti-HBc and/or positive for anti-HBs after resolution of infection, while 123 uninfected subjects were all negative for HBsAg, anti-HBc and anti-HBs. All samples in this study were collected from subjects who have lived at the same area in Thailand, suggesting that the genetic background would be balanced between a case and control.

### Genotyping assays

DNA was extracted from peripheral blood mononuclear cell using phenol-chloroform DNA extraction. The concentration of DNA was determined by NanoDrop 2000c spectrophotometer (Thermo Scientific, Wilmington, DE). We determined SNPs of *HLA-DPA1* (rs3077), *HLA-DPB1* (rs9277378 and rs3128917), and the genes *TCF19* (rs1419881) and *EHMT2* (rs652888) by commercial TaqMan PCR assays (Applied Biosystems, USA). In this study we investigated *HLA-DPB1* (rs9277378) because this SNP had a high level of linkage disequilibrium with rs9277535 (D′ = 1.00, R^2^ = 0.954) [20] and was clearly detectable by the TaqMan assay rather than rs9277535.

### Statistical analyses

In this study, Hardy-Weinberg equilibrium was performed on each SNP. The Chi-square test of independence and Odds Ratio (OR) from two-by-two tables for comparisons between case and control groups was performed using Microsoft Excel. Statistical significance was defined by *P*<0.05. The calculated of possibility level was established using Chi-square contingency table analysis.

## Results

Subjects were defined into 4 groups: group 1) HCC (age = 58.2±12 years, 190/230 (82.6%) male); group 2) CHB (age = 46.6±10 years, 144/219 (65.7%) male); group 3) those with resolved HBV (age = 48.2±6 years, 83/113 (73.5%) male); and group 4) HBV uninfected subjects (age = 46.7±6 years, 73/123 (59.3%) male). The details are given in Table 1. To find the genetic factor associated with chronicity of HBV infection, however, the two groups (group 1 and 2) were combined (designated “HBV carriers”). Indeed, according to the frequencies of minor alleles of the SNPs in the *HLA-DP*, *TCF19* and *EHMT2* genes listed in Table 2, the frequencies of minor alleles of these 5 SNPs in HCC and CHB were similar (data shown in Table S1). The composite HBV carriers group had a minor allele frequency for rs3077 and rs9277378 lower than in groups 3 and 4 (OR = 0.57, 95% CI = 0.42–0.78, *p*<0.001 and OR = 0.63, 95% CI = 0.47–0.85, *p* = 0.008 for rs3077, OR = 0.59, 95% CI = 0.44–0.81, *p* = 0.001 and OR = 0.56, 95% CI = 0.42–0.75, *p*<0.001 for rs9277378, respectively). In contrast, the minor allele frequency for rs1419881 in HBV carriers was similar to group 3 (OR = 0.80, 95% CI = 0.60–1.08, *p* = 0.142) but lower than in group 4 (OR = 0.64, 95% CI = 0.48–0.85, *p* = 0.002). Moreover, minor allele frequency for rs3128917 and rs652888 in HBV carriers was comparable to groups 3 and 4 (OR = 1.14, 95% CI = 0.85–1.53, *p* = 0.371 and OR = 1.06, 95% CI = 0.80–1.41, *p* = 0.673 for rs3128917; OR = 1.14, 95% CI = 0.84–1.55, *p* = 0.400 and OR = 1.12, 95% CI = 0.83–1.50, *p* = 0.471 for rs652888, respectively).

**Table 1:** Characteristics of participants in HCC, CHB, resolved HBV and HBV uninfected subjects in Thailand.

	HCC (n = 230)	CHB^a^ (n = 219)	Resolved^b^ (n = 113)	Uninfected^c^ (n = 123)
Age (years)	58.2±12	46.6±10	48.2±6	46.7±6
Male	190 (82.6%)	144 (65.7%)	83 (73.5%)	73 (59.3%)
HBsAg positive	230 (100%)	219 (100%)	0	0
ALT>40 (IU/L)	43 (18.7%)	61 (27.8%)	-	-
Alb (g/dl)	3.7 (2.5–5.6)	4.5 (3–5.2)	-	-
TB (mg/dl)	1.2 (0.17–14.8)	0.56 (0.2–2.67)	-	-

Abbreviation: HCC, hepatocellular carcinoma; CHB, chronic hepatitis B; HBsAg, hepatitis B surface antigen;

ALT, Alanine transaminase; Alb, Albumin; TB, Total bilirubin.

a Defined as chronic hepatitis B includes chronic HBV infection but not cirrhosis and HCC.

b Defined as HBsAg negative but anti-HBc or/and anti-HBs positive.

c Defined as any HBV serological markers negative.

**Table 2:** Minor allele frequencies in HBV carriers, resolved HBV and uninfected subjects in Thailand.

						HBV carriers vs. Resolved		HBV carriers vs. Uninfected	
SNPs	Gene	Minor alleles^a^	HBV carriers^b^ (2n = 898)	Resolved (2n = 226)	Uninfected (2n = 246)	OR (95% CI)	*P* values	OR (95% CI)	*P* values
rs3077	*HLA-DPA1*	T	227 (25.3%)	84 (37.2%)	86 (35.0%)	0.57 (0.42–0.78)	<0.001	0.63 (0.47–0.85)	0.008
rs9277378	*HLA-DPB1*	A	237 (26.4%)	85 (37.6%)	96 (39.0%)	0.59 (0.44–0.81)	0.001	0.56 (0.42–0.75)	<0.001
rs3128917	*HLA-DPB1*	G	459 (51.1%)	108 (47.8%)	122 (49.6%)	1.14 (0.85–1.53)	0.372	1.06 (0.80–1.41)	0.673
rs1419881	*TCF19*	C	361 (40.2%)	103 (45.6%)	126 (51.2%)	0.80 (0.60–1.08)	0.142	0.64 (0.48–0.85)	0.002
rs652888	*EHMT2*	C	329 (36.6%)	76 (33.6%)	84 (34.1%)	1.14 (0.84–1.55)	0.400	1.11 (0.83–1.50)	0.478

Abbreviation: CI, confidence interval; OR, odds ratio.

a Defined by using data from public database (NCBI).

b Defined as the combination between HCC and CHB.

The results of Hardy-Weinberg equilibrium analysis of each SNPs were shown in Table 3. All data were over 0.01 (*p*>0.01), indicating that the frequencies did not deviate from Hardy-Weinberg equilibrium. The genotype distribution in HBV carriers compared to subjects with HBV resolution showed that both rs3077 and rs9277378 were significantly associated with protective effects against CHB in minor dominant model (OR = 0.45, 95% CI = 0.30–0.69, *p*<0.001 for rs3077 and OR = 0.47, 95% CI = 0.31–0.72, *p*<0.001 for rs9277378, are described in Table 3), suggesting that major homozygous genotypes were risk factors with the chronicity of HBV. The other SNPs rs3128917, rs1419881 and rs652888 were not associated against HBV carrier status (OR = 1.22, 95% CI = 0.76–1.97, *p* = 0.413 for rs3128917, OR = 0.67, 95% CI = 0.42–1.06, *p* = 0.084 for rs1419881 and OR = 1.31, 95% CI = 0.87–2.00, *p* = 0.198 for rs652888, respectively).

**Table 3:** Genotype frequencies in HBV carriers, resolved HBV and uninfected subjects in Thailand.

					HBV carriers vs. Resolved		HBV carriers vs. Uninfected	
SNP	Genotype	HBV carriers^a^ (n = 449)	Resolved (n = 113)	Uninfected (n = 123)	OR (95% CI)	*P* values	OR (95% CI)	*P* values
rs3077	CC	259 (57.7%)	43 (38.1%)	57 (46.3%)	1.00	-	1.00	-
*HLA-DPA1*	CT	153 (34.1%)	56 (49.6%)	46 (37.4%)	0.45 (0.29–0.71)	<0.001	0.73 (0.47–1.13)	0.161
	TT	37 (8.2%)	14 (12.4%)	20 (16.3%)	0.44 (0.22–0.88)	0.018	0.41 (0.22–0.75)	0.003
	**Dominant** b				**0.45 (0.30–0.69)**	**<0.001**	**0.63 (0.42–0.95)**	**0.025**
	HWEp	0.038	0.516	0.049				
rs9277378	GG	242 (53.9%)	40 (35.4%)	48 (39.0%)	1.00	-	1.00	-
*HLA-DPB1*	AG	177 (39.4%)	61 (54.0%)	54 (43.9%)	0.48 (0.31–0.75)	0.001	0.65 (0.42–1.00)	0.051
	AA	30 (6.7%)	12 (10.6%)	21 (17.1%)	0.41 (0.20–0.87)	0.018	0.28 (0.15–0.54)	<0.001
	**Dominant**				**0.47 (0.31–0.72)**	**<0.001**	**0.55 (0.36–0.82)**	**0.003**
	HWEp	0.757	0.110	0.390				
rs3128917	TT	99 (22.0%)	29 (25.7%)	38 (30.9%)	1.00	-	1.00	-
*HLA-DPB1*	TG	241 (53.7%)	60 (53.1%)	48 (39.0%)	1.18 (0.71–1.94)	0.525	1.93 (1.19–3.13)	0.008
	GG	109 (24.3%)	24 (21.2%)	37 (30.1%)	1.33 (0.73–2.44)	0.355	1.13 (0.67–1.92)	0.648
	**Dominant**				**1.22 (0.76–1.97)**	**0.413**	**1.58 (1.02–2.46)**	**0.042**
	HWEp	0.117	0.496	0.015				
rs1419881	TT	162 (36.1%)	31 (27.4%)	30 (24.4%)	1.00	-	1.00	-
*TCF19*	TC	213 (47.4%)	61 (54.0%)	60 (48.8%)	0.67 (0.41–1.08)	0.097	0.66 (0.41–1.07)	0.088
	CC	74 (16.5%)	21 (18.6%)	33 (26.8%)	0.67 (0.36–1.25)	0.210	0.42 (0.24–0.73)	0.002
	**Dominant**				**0.67 (0.42–1.06)**	**0.084**	**0.57 (0.36–0.90)**	**0.015**
	HWEp	0.778	0.349	0.792				
rs652888	TT	169 (37.6%)	50 (44.2%)	57 (46.3%)	1.00	-	1.00	-
*EHMT2*	TC	231 (51.4%)	50 (44.2%)	48 (39.0%)	1.37 (0.88–2.12)	0.162	1.62 (1.05–2.50)	0.027
	CC	49 (10.9%)	13 (11.5%)	18 (14.6%)	1.12 (0.56–2.22)	0.756	0.92 (0.49–1.70)	<0.001
	**Dominant**				**1.31 (0.87–2.00)**	**0.198**	**1.09 (0.65–1.82)**	**0.080**
	HWEp	0.022	0.926	0.142				

Abbreviation: CI, confidence interval; OR, odds ratio ; HWEp, Hardy-Weinberg equilibrium analysis.

a Defined as the combination between HCC and CHB.

b Defined as a minor dominant according to the comparison between heterozygous+minor homozygous genotype and major homozygous genotype (*eg.* rs3077; CT+TT vs. CC).

The genotype frequencies for 5 SNPs are shown in Table 3. Comparing HBV carriers with uninfected subjects showed that rs3077, rs9277378 and rs1419881 were all protectively associated with chronic HBV infection (OR = 0.63, 95% CI = 0.42–0.95, *p* = 0.025 for rs3077 and OR = 0.55, 95% CI = 0.36–0.82, *p* = 0.003 for rs9277378 and OR = 0.57, 95% CI = 0.36–0.90, *p* = 0.015 for rs1419881, respectively). Comparing HBV carriers and uninfected subjects rather than those with resolved infection regarding rs1419881 was significantly protective association against CHB, but rs3128917 and rs652888 were not associated against CHB (OR = 1.58, 95% CI = 1.02–2.46, *p* = 0.042 for rs3128917 and OR = 1.09, 95% CI = 0.65–1.82, *p* = 0.080 for rs652888). When we consider the Bonferroni corrections (5 SNPs), however, the P value for rs1419881 did not reach the level of significant difference (0.015>0.05/5) between HBV carriers and HBV uninfected subjects. These data suggested that other SNPs, rs1419881, rs3128917 and rs652888 were not associated with HBV carriers in this study.

Results of meta-analysis for 3 SNPs (rs3077, rs9277378 and rs3128917) in the *HLA* gene were shown in Table S2 and S3; HBV carriers were compared to HBV resolved or HBV uninfected subjects, respectively. While the other 2 SNPs were published only from Korean population, thus the meta-analysis appeared only between HBV carriers and HBV uninfected subjects. All SNPs analyzed by the meta-analysis were significantly associated with HBV carriers.

The associations between these 5 SNPs and HBV status are depicted graphically in Figure S1. Each histogram compares HBV carriers with subjects that have resolved HBV infection or were never infected. The results showed that the minor dominant model of rs3077 and rs9277378 was highly protective associated against chronic HBV, while no significant associations were observed with rs3128917 and rs652888. Furthermore, comparing the frequency of rs1419881 between HBV carriers and uninfected subjects also revealed its association against chronic HBV infection but the association with resolved HBV did not achieve statistical significance.

## Discussion

Genetic variations of rs3077 and rs9277378, but not rs3128917, rs1419881 and rs652888, were significantly associated with HBV carriers relative to resolved HBV in Thai population. In the human genome, single nucleotide polymorphisms are found in every 300–570 nucleotides. Many SNPs have no effect on the function of the encoded proteins, but some variants do appear in regulatory or coding part of the gene and affect gene expression level or protein function which can give rise to disease [21] such as the 3 SNPs including rs3077, rs9277378 and rs3128917 in *HLA-DP* region of MHC class II. The function of HLA-DP is to present bound peptide antigens, e.g. from HBV, at the surface of antigen-presenting cells. CD4+ T cells recognize these antigens and initiate the adaptive immune response. They assist the MHC class I-restricted CD8+ T cells which are the primary cellular effectors mediating HBV clearance from the liver during acute viral infection [22]. HBV infection will either be cleared by these means, or establish itself as a chronic infection. The reason for the latter is unclear but may be related to variation of *HLA-DP* alleles. Thus, the position of *HLA-DP* SNPs might be associated with possibility of clearance or chronicity. The rs3077 and rs9277535 SNPs are located within the 3′ untranslated region (UTR) of *HLA-DPA1* and *HLA-DPB1*, respectively while rs3128917 is located downstream of *HLA-DPB1*.

Recent investigations have identified 11 risk alleles for CHB related to mRNA expression of *HLA-DPA1* and *HLA-DPB1* [23]. The results showed that only these two alleles, rs3077 and rs9277535 were strongly associated with the risk of CHB and decreased expression of *HLA-DPA1* and *HLA-DPB1*, respectively. In contrast, while rs3128917 was associated with CHB, it was not associated with the level of HLA-DPB1 expression [23]. Variation at 5′ and 3′ UTRs can alter the binding sites of regulatory proteins which protect and stabilize newly synthesized RNA, either increasing or decreasing binding [24], [25]. Nevertheless, the present study showed that rs3128917 was not associated with HBV carrier status in Thailand. Because rs3128917 is located downstream of the direction of transcription of the gene, this suggests that it does not affect regulation or coding of the gene and would have no effect on HLA protein expression.

The results from the present study not only establish the importance of variation at the *HLA-DP* gene but also explore two new SNPs, rs1419881 located in *TCF19* and rs652888 in the *EHMT2* gene [16]. *TCF19* (or transcription factor SC1) is a late growth regulatory gene like histone, thymidine kinase etc, maximally expressed at the onset of DNA synthesis at the G1-S boundary and S phase of cell cycle. This protein is also involved in regulations of growth and transcription factors controlling the number and development of peripheral-blood monocytes and erythrocytes [26]. The *EHMT2* gene is a histone methyltransferase [18] mainly responsible for mono- and di-methylation of H3K9 in euchromatin. This changes the conformation of chromatin from euchromatin to heterochromatin and then affects gene repression [19]. Histone methylation has a critical role in gene transcription and epigenetic events [27]–[30].

According to recently published GWAS data [11], two SNPs associated with the risk for CHB in the Korea population were identified. These were the top signals in the genome-wide significance level analysis and were independently associated with *HLA-DP* and *HLA-DQ*, respectively. The authors then confirmed the results in a replication sample, showing that the frequency of their two SNPs strongly associated with CHB; OR = 0.76, 95% CI = 0.68–0.86, *p* = 4.51E-11 for rs1419881 and OR = 1.26, 95% CI = 1.07–1.47, *p* = 2.78E-06 for rs652888 [16]. Furthermore, another GWAS study focused on HLA, of hepatitis B vaccinated people in Indonesia, showed that rs652888 was also associated with risk of CHB (*p*≤0.0001) in that population [31].

In the present study, however, we found that rs1419881 tended to be associated with chronic HBV infection, based on the results of a comparison between HBV carriers and uninfected subjects. Nonetheless, it did not reach the significance by the Bonferroni corrections, as well as when HBV carriers were compared with patients who had their HBV infection resolved, no association with rs1419881 was observed. The second SNP, rs652888, was not associated with chronic HBV infection in the Thai population. Although our study had sampling error due to small samples, it might be another effect that the result between rs652888 in *EHMT2* gene and chronic hepatitis B in Thai population was not associated. The reason for these negative findings for the two SNPs might be due to the affected gene functions that were not involved with the immune system or processes of persistent infection. Data supporting this notion are to be found in the GWAS data for the Korean population, where pathway analysis of genes involved in the regulation of immune function showed that *TCF19* and *EHMT2* genes are not significantly involved in human immunity [16].

Mapping the position of the two new SNPs showed that rs1419881 located at the 3′ UTR of exon 4, with a tendency towards association with CHB and rs652888 which is not associated with CHB located on an intron. The position of each SNP might affect the phenotype of gene expression and susceptibility to disease, explaining why some are associated with chronic HBV infection, and others not. According to previous publications, the 3′ UTR of the *HLA-DP* region is strongly involved with regulating HLA-DP expression and influences the outcome of HBV infection [32]. In addition, another study showed that variation of the 3′ UTR of HLA-C was strongly associated with HLA-C expression levels and with control of human immunodeficiency virus [33]. This illustrated the general principle that the position of SNPs affects association with diseases.

The prevalence of HBV in Eastern countries, i.e. Asia, sub-Saharan Africa and the Pacific is much higher than in Western Europe and America. Most people in Eastern countries are infected with HBV during childhood and 8–10% of these develop CHB. In contract, the frequency of chronic carriers in Western Europe and North America is ≤1%. Furthermore, previous GWAS and meta-analysis reported that A alleles at rs3077 and rs9277353 have protective effects against CHB. Asian and African populations, especially Chinese, have lower frequencies of A alleles than European and American populations [10], [34], [35]. Moreover, the previous study showed no associations of rs3077 and rs9277535 with progressive CHB infection; however rs3077 was highly significant associated with HBV infection but not associated with rs9277353 in Caucasian populations [36].

While the frequency of alleles at rs3128917 and rs1419881 in Asian and African populations are quite similar, Northern and Western European populations have high frequencies of the protective T allele at rs3128917 but have low T allele frequencies (a risk allele for CHB) at rs1419881. The allele frequencies of populations in the worldwide for conspicuous details came from dbSNP Short Genetic Variations available at http://www.ncbi.nlm.nih.gov/projects/SNP/snp_ref.cgi. Lastly, both ethnic Eastern and Western populations have similar allele frequencies at rs652888, carrying a risk for CHB, with T allele frequencies very much higher than C allele frequencies, which has a protective effect. In addition, evolution of genomic characteristics, the migratory history of different populations, as well as HBV genotypes [37], HBV carrier rate [38] and pathological procession of liver disease [39] in each country may affect the distribution of *HLA* alleles. This was illustrated by a recent report in two Han Chinese populations (southern and northern) having different distributions of *HLA-DP* genes [39]. Thus, the genetics of the host is one of the factors influencing and predicting disease outcome [40].

According to less number of samples, it might influence statistical power in this study. Thus, we made another statistic meta-analysis of data obtained from previous reports and this study in Table S3. We compared HBV carriers with HBV uninfected subjects, because most previous studies also compared CHB with HBV clearance and/or healthy (negative for any HBV serological markers). Interestingly, all SNPs analyzed by the meta-analysis were significantly associated with HBV carriers. These results could support our data in Thailand. Additionally, no heterogeneity was observed between HBV carriers and HBV-resolved subjects (P_het_ = 0.10 for rs3077, 0.79 for rs9277378, and 0.07 for rs3128917), as well as between HBV carriers and HBV uninfected subjects (P_het_ = 0.10 for rs3077, 0.02 for rs9277378, 0.91 for rs1419881, and 0.04 for rs652888) except for rs9277378 (P_het_ = 0.000), for the minor allele frequency (MAF) of only rs9277378 was different between HapMap-CHB (MAF = 46.3% of G allele) and HapMap-JPT (MAF = 44.8% of T allele).

In the present study, we determined associations of variations at the *HLA-DP* gene with outcome in HBV infected Thai patients and the major homozygous genotypes of rs3077 and rs9277378, but not rs3128917, were significantly associated with HBV carrier status. Although genetic variation of two new SNPs, rs1419881 in the *TCF19* gene and rs652888 in the *EHMT2* gene, were not associated with the outcome of HBV infection in the Thai population, a large-scale study should be required.

## Supporting Information

Figure S1 **Association of 5 SNPs with HBV carriers, resolved HBV and uninfected subjects in Thailand.** The results were compared between percentages of combination of heterozygous genotypes and minor homozygous genotypes (White square) with percentages of major homozygous genotypes (Grey square). Five SNPs applied in this study were rs3077, rs9277378 and rs3128917 in *HLA-DP* gene, rs1419881 in *TCF19* gene and rs652888 in *EHMT2* gene. OR, odds ratio; (lower-upper), 95% confidence interval.
(PPTX)

Table S1 **Minor allele frequencies in HCC, CHB, resolved HBV and uninfected subjects in Thailand.**
(DOC)

Table S2 **The meta-analysis of minor allele frequencies in HBV carriers and resolved HBV.**
(DOC)

Table S3 **The meta-analysis of minor allele frequencies in HBV carriers and uninfected subject.**
(DOC)
