# Supplementary material for: Genetic Association of Human Leukocyte Antigens with Chronicity or Resolution of Hepatitis B Infection in Thai Population
Source: PLoS One. 2014 Jan 23;9(1):e86007. doi: 10.1371/journal.pone.0086007 (PMC3900446; doi:10.1371/journal.pone.0086007)
Supplement: Figure S1 — Association of 5 SNPs with HBV carriers, resolved HBV and uninfected subjects in Thailand. The results were compared between percentages of combination of heterozygous genotypes and minor homozygous genotypes (White square) with percentages of major homozygous genotypes (Grey square). Five SNPs applied in this study were rs3077, rs9277378 and rs3128917 in HLA-DP gene, rs1419881 in TCF19 gene and rs652888 in EHMT2 gene. OR, odds ratio; (lower-upper), 95% confidence interval. (PPTX) [file pone.0086007.s001.pptx]

## Slide 1
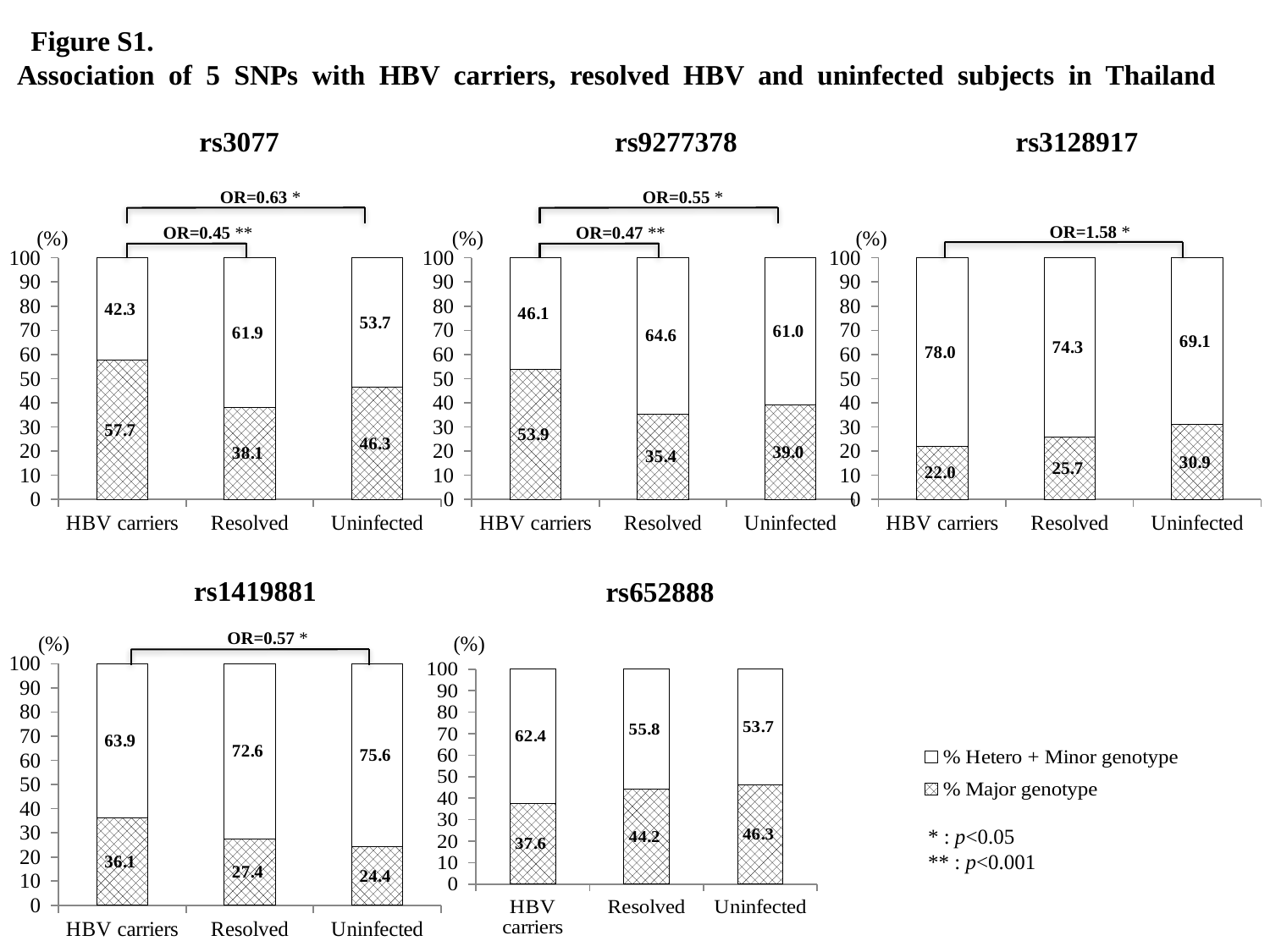

# Figure S1. Association of 5 SNPs with HBV carriers, resolved HBV and uninfected subjects in Thailand
rs3077
rs9277378
rs3128917
OR=0.63 *
OR=0.55 *
OR=1.58 *
OR=0.45 **
OR=0.47 **
(%)
(%)
(%)
### Chart
| Category | MA | HE + MI |
|---|---|---|
| HBV carriers | 57.6837416481069 | 42.31625835189308 |
| Resolved | 38.05309734513274 | 61.946902654867245 |
| Uninfected | 46.341463414634134 | 53.65853658536586 |
### Chart
| Category | MA | HE + MI |
|---|---|---|
| HBV carriers | 53.89755011135856 | 46.102449888641424 |
| Resolved | 35.39823008849557 | 64.60176991150442 |
| Uninfected | 39.02439024390244 | 60.97560975609755 |
### Chart
| Category | MA | HE + MI |
|---|---|---|
| HBV carriers | 22.048997772828507 | 77.9510022271715 |
| Resolved | 25.663716814159287 | 74.33628318584073 |
| Uninfected | 30.89430894308943 | 69.10569105691056 |rs1419881
rs652888
OR=0.57 *
(%)
(%)
### Chart
| Category | MA | HE + MI |
|---|---|---|
| HBV carriers | 36.080178173719375 | 63.919821826280604 |
| Resolved | 27.43362831858407 | 72.56637168141593 |
| Uninfected | 24.39024390243902 | 75.60975609756095 |
### Chart
| Category | % Major genotype | % Hetero + Minor genotype |
|---|---|---|
| HBV carriers | 37.63919821826281 | 62.36080178173719 |
| Resolved | 44.247787610619454 | 55.75221238938053 |
| Uninfected | 46.341463414634134 | 53.65853658536586 |* : p<0.05
** : p<0.001
