# Supplementary material for: Genetic Association of Human Leukocyte Antigens with Chronicity or Resolution of Hepatitis B Infection in Thai Population
Source: PLoS One. 2014 Jan 23;9(1):e86007. doi: 10.1371/journal.pone.0086007 (PMC3900446; doi:10.1371/journal.pone.0086007)
Supplement: Table S1 — Minor allele frequencies in HCC, CHB, resolved HBV and uninfected subjects in Thailand. (DOC) [file pone.0086007.s002.doc]

|  |  |  |  |  |  | HCC vs. CHB | | HCC vs. Resolved | | HCC vs. Uninfected | | CHB vs. Resolved | | CHB vs. Uninfected | |
| --- | --- | --- | --- | --- | --- | --- | --- | --- | --- | --- | --- | --- | --- | --- | --- |
| SNPs | Minor alleles^a^ | HCC (2n=460) | CHB (2n=438) | Resolved (2n=226) | Uninfected (2n=246) | OR (95% CI) | *P* values | OR (95% CI) | *P* values | OR (95% CI) | *P* values | OR (95% CI) | *P* values | OR (95% CI) | *P* values |
| rs3077 | T | 128 (27.8%) | 99 (22.6%) | 84 (37.2%) | 86 (35.0%) | 1.32 (0.98-1.79) | 0.072 | 0.65 (0.46-0.91) | 0.013 | 0.72 (0.51-1.00) | 0.049 | 0.49 (0.35-0.70) | <0.001 | 0.54  (0.38-0.77) | <0.001 |
| rs9277378 | A | 136 (29.6%) | 101 (23.1%) | 85 (37.6%) | 96 (39.0%) | 1.40 (1.04-1.89) | 0.027 | 0.70 (0.50-0.97) | 0.034 | 0.66 (0.47-0.91) | 0.011 | 0.50 (0.35-0.70) | <0.001 | 0.47  (0.33-0.66) | <0.001 |
| rs3128917 | G | 218 (47.4%) | 241 (55.0%) | 108 (47.8%) | 122 (49.6%) | 0.74 (0.57-0.96) | 0.022 | 0.98 (0.72-1.35) | 0.922 | 0.92 (0.67-1.25) | 0.577 | 1.34 (0.97-1.84) | 0.077 | 1.24  (0.91-1.70) | 0.172 |
| rs1419881 | C | 192 (41.7%) | 169 (38.6%) | 103 (45.6%) | 126 (51.2%) | 1.14 (0.87-1.49) | 0.335 | 0.86 (0.62-1.18) | 0.340 | 0.68 (0.5-0.93) | 0.680 | 0.60 (0.44-0.82) | 0.001 | 1.25  (0.87-1.80) | 0.22 |
| rs652888 | C | 169 (36.7%) | 160 (36.5%) | 76 (33.6%) | 84 (34.1%) | 1.01 (0.77-1.32) | 0.948 | 1.15 (0.82-1.60) | 0.424 | 1.12 (0.81-1.55) | 0.494 | 1.14 (0.81-159) | 0.459 | 1.11  (0.80-1.54) | 0.532 |

**Table S1. Minor allele frequencies in HCC, CHB, resolved HBV and uninfected subjects in Thailand**

Abbreviation: CI, confidence interval; OR, odds ratio

^a^Defined by using data from public database (NCBI)
