# Supplementary material for: Genetic Association of Human Leukocyte Antigens with Chronicity or Resolution of Hepatitis B Infection in Thai Population
Source: PLoS One. 2014 Jan 23;9(1):e86007. doi: 10.1371/journal.pone.0086007 (PMC3900446; doi:10.1371/journal.pone.0086007)
Supplement: Table S2 — The meta-analysis of minor allele frequencies in HBV carriers and resolved HBV. (DOC) [file pone.0086007.s003.doc]

**Table S2. The meta-analysis of minor allele frequencies in HBV carriers and resolved HBV**

|  |  |  |  |  | HBV carriers vs. Resolved | |
| --- | --- | --- | --- | --- | --- | --- |
| SNPs | Minor alleles^a^ | Population | HBV carriers | Resolved | OR (95% CI) | *P* values |
| rs3077 | T | Thai (This study) | 227 (25.3%) | 84 (37.2%) | 0.57 (0.42-0.78) | <0.001 |
|  |  | Japanese (Ref 14) | 224 (25.7%) | 244 (37.1%) | 0.59 (0.47-0.73) | <0.001 |
|  |  | Han Chinese (Ref 12) | 414 (28.1%) | 669 (42.8%) | 0.52 (0.45-0.61) | <0.001 |
|  |  | Zhuang Chinese (Ref 12) | 70 (19.8%) | 98 (23.6%) | 0.80 (0.57-1.13) | 0.199 |
|  |  | Han Chinese (Ref 13) | 711 (29.2%) | 242 (42.2%) | 0.57 (0.47-0.68) | <0.001 |
|  |  | Han Chinese (Ref 15) | 1,420 (27.0%) | 856 (32.2%) | 0.78 (0.70-0.86) | <0.001 |
|  |  | Hong Kong (Ref 40) | 207 (20.7%) | 143 (27.6%) | 0.68 (0.54-0.88) | 0.002 |
|  |  | Korean (Ref 14) | 127 (29.5%) | 106 (50.5%) | 0.41 (0.29-0.58) | <0.001 |
|  |  | **Meta analysis** |  |  | **0.65 (0.61-0.70)** | **<0.001** |
| rs9277378 | A | Thai (This study) | 237 (26.4%) | 85 (37.6%) | 0.59 (0.44-0.81) | 0.001 |
|  |  | Han Chinese (Ref 12) | 528 (35.9%) | 698 (44.6%) | 0.70 (0.60-0.80) | <0.001 |
|  |  | Zhuang Chinese (Ref 12) | 83 (23.4%) | 141 (33.9%) | 0.60 (0.43-0.82) | 0.001 |
|  |  | Han Chinese (Ref 15) | 1,797 (34.2%) | 1,166 (43.6%) | 0.67 (0.61-0.74) | <0.001 |
|  |  | Hong Kong (Ref 40) | 242 (24.2%) | 176 (34.0%) | 0.62 (0.49-0.78) | <0.001 |
|  |  | **Meta analysis** |  |  | **0.66 (0.61-0.70)** | **<0.001** |
| rs3128917 | G | Thai (This study) | 459 (51.1%) | 108 (47.8%) | 1.14 (0.85-1.53) | 0.372 |
|  |  | Hong Kong (Ref 40) | 665 (66.5%) | 287 (55.4%) | 1.60 (1.29-1.99) | <0.001 |
|  |  | **Meta analysis** |  |  | **1.28 (1.08-1.52)** | **0.004** |
| rs1419881 | C | Thai (This study) | 361 (40.2%) | 103 (45.6%) | 0.80 (0.60-1.08) | 0.142 |
| rs652888 | C | Thai (This study) | 329 (36.6%) | 76 (33.6%) | 1.14 (0.84-1.55) | 0.400 |

Abbreviation: CI, confidence interval; OR, odds ratio; Ref, reference number in the text

^a^Defined by using data from public database (NCBI)
