# Supplementary material for: Genetic Association of Human Leukocyte Antigens with Chronicity or Resolution of Hepatitis B Infection in Thai Population
Source: PLoS One. 2014 Jan 23;9(1):e86007. doi: 10.1371/journal.pone.0086007 (PMC3900446; doi:10.1371/journal.pone.0086007)
Supplement: Table S3 — The meta-analysis of minor allele frequencies in HBV carriers and uninfected subject. (DOC) [file pone.0086007.s004.doc]

**Table S3. The meta-analysis of minor allele frequencies in HBV carriers and uninfected subject**

|  |  |  |  |  | HBV carriers vs. Uninfected | |
| --- | --- | --- | --- | --- | --- | --- |
| SNPs | Minor alleles^a^ | Population | HBV carriers | Uninfected | OR (95% CI) | *P* values |
| rs3077 | T | Thai (This study) | 227 (25.3%) | 86 (35.0%) | 0.63 (0.47-0.85) | 0.003 |
|  |  | Japanese (Ref 10) | 999 (26.4%) | 2,624 (39.0%) | 0.56 (0.51–0.61) | <0.001 |
|  |  | Japanese (Ref 11) | 1,427 (26.7%) | 5,232 (40.3%) | 0.54 (0.50-0.58) | <0.001 |
|  |  | Japanese (Ref 14 ) | 224 (25.7%) | 361 (43.1%) | 0.46 (0.37-0.56) | <0.001 |
|  |  | Han Chinese (Ref 13) | 711 (29.2%) | 173 (38.1%) | 0.67 (0.54-0.82) | <0.001 |
|  |  | Hong Kong (Ref 40) | 207 (20.7%) | 141 (28.8%) | 0.65 (0.50-0.83) | 0.001 |
|  |  | Caucasian (Ref 36) | 305 (75.9%) | 365 (82.2%) | 0.68 (0.49-0.95) | 0.023 |
|  |  | Korean (Ref 14) | 127 (29.5%) | 136 (46.9%) | 0.47 (0.35-0.65) | <0.001 |
|  |  | Korean (Ref 16) | 790 (28.8%) | 2,580 (43.9%) | 0.52 (0.47-0.57) | <0.001 |
|  |  | **Meta analysis** |  |  | **0.55 (0.53-0.58)** | **<0.001** |
| rs9277378 | A | Thai (This study) | 237 (26.4%) | 96 (39.0%) | 0.56 (0.42-0.75) | <0.001 |
|  |  | Japanese (Ref 10) | 1,118 (29.6%) | 2,854 (42.6%) | 0.57 (0.52-0.62) | <0.001 |
|  |  | Japanese (Ref 11) | 1,588 (29.8%) | 5,588 (43.0%) | 0.56 (0.52-0.60) | <0.001 |
|  |  | Hong Kong (Ref 40) | 242 (24.2%) | 159 (32.4%) | 0.66 (0.52-0.84) | 0.001 |
|  |  | Caucasian (Ref 36) | 298 (74.1%) | 356 (75.7%) | 0.92 (0.67-1.25) | 0.583 |
|  |  | Korean (Ref 16) | 908 (33.1%) | 2,844 (48.4%) | 0.53 (0.48-0.58) | <0.001 |
|  |  | **Meta analysis** |  |  | **0.56 (0.54-0.59)** | **<0.001** |
| rs3128917 | G | Thai (This study) | 459 (51.1%) | 122 (49.6%) | 1.06 (0.80-1.41) | 0.673 |
|  |  | Japanese (Ref 11) | 286 (31.2%) | 1,877 (45.6%) | 0.54 (0.46-0.63) | <0.001 |
|  |  | Hong Kong (Ref 40) | 665 (66.5%) | 288 (58.8%) | 1.39 (1.11-1.74) | 0.004 |
|  |  | **Meta analysis** |  |  | **0.77 (0.69-0.86)** | **<0.001** |
| rs1419881 | C | Thai (This study) | 361 (40.2%) | 126 (51.2%) | 0.64 (0.48-0.85) | 0.002 |
|  |  | Korean (Ref 16) | 913 (33.3%) | 2,550 (43.4%) | 0.65 (0.59-0.72) | <0.001 |
|  |  | **Meta analysis** |  |  | **0.65 (0.59-0.71)** | **<0.001** |
| rs652888 | C | Thai (This study) | 329 (36.6%) | 84 (34.1%) | 1.11 (0.83-1.50) | 0.478 |
|  |  | Korean (Ref 16) | 543 (19.8%) | 805 (13.7%) | 1.56 (1.38-1.75) | <0.001 |
|  |  | **Meta analysis** |  |  | **1.48 (1.32-1.66)** | **<0.001** |

Abbreviation: CI, confidence interval; OR, odds ratio; Ref, reference number in the text

^a^Defined by using data from public database (NCBI)
